# Energy Harvesting Technologies and Applications for the Internet of Things and Wireless Sensor Networks

**DOI:** 10.3390/s24144688

**Published:** 2024-07-19

**Authors:** Slim Naifar, Olfa Kanoun, Carlo Trigona

**Affiliations:** 1 Professorship of Measurement and Sensor Technology, Department of Electrical Engineering and Information Technology, Chemnitz University of Technology, 09126 Chemnitz, Germany; 2 Laboratory of Electromechanical Systems, National Engineering School, University of Sfax, Sfax 3038, Tunisia; 3 Dipartimento di Ingegneria Elettrica, Elettronica e Informatica, University of Catania, Viale Andrea Doria 6, 95125 Catania, Italy; carlo.trigona@dieei.unict.it

The field of Internet of Things (IoT) technologies is advancing rapidly, driven by the critical need for autonomous and sustainable wireless sensor networks [1,2,3,4,5,6]. This growth has been accompanied by increased emphasis on energy harvesting (EH) techniques, which aim to develop technologies for capturing and converting ambient energy into usable electrical power. These methods leverage various environmental energy sources, converting them into electrical energy through appropriate transduction mechanisms and energy management circuits. The use of ambient energy sources such as solar, thermal, vibrational, and radio frequency energy has led to innovations in energy harvesting systems, incorporating advanced materials and novel transduction methods, significantly contributing to the development of efficient and sustainable wireless sensor nodes [7,8,9,10,11,12,13,14]. Research in this field also emphasizes minimizing the energy consumption of wireless sensor nodes, thereby enhancing the efficiency and sustainability of Wireless Sensor Networks (WSNs) [15,16,17,18].

The following Special Issue compiles 31 papers that offer a comprehensive overview of recent advancements in energy harvesting technologies and their integration into IoT systems. The collection highlights key findings and their implications for future research and applications. Included are studies covering energy harvesting devices, their optimization and potential applications (Contributions 1–9), piezoelectric and pyroelectric nanogenerators (Contributions 10–11), as well as investigations into thermoelectric generators and solar harvester modules (Contributions 12–14). Additionally, this Special Issue features research on the design and applications of radio frequency harvesters (Contributions 15–19), wireless power transfer technologies (Contributions 20–21), and the design, implementation, and evaluation of wireless-powered communication networks (Contributions 22–30). A comprehensive review paper is also included, focusing on aging mechanisms and their impact on the electrical performance of e-textiles (Contribution 31).

In Contribution 1, research is conducted on a vibration-based cantilever beam of composite-laminated piezoelectric patches through an experimental study of its characteristics and a modeling study of energy harvesting. The experimental study investigates the harvesting capacity and electromechanical characteristics of the cantilever harvester. In Contribution 2, a pendulum transducer prototype is tested with an online hydrometric measurement station. The system is successfully validated through experimental studies in a river. In Contribution 3, process optimization of key parameters such as beam spacing, flux density, and optimal impedance load matching of magnetic coupled piezoelectric harvesters is presented. The analysis of calculated voltage outputs based on theoretical and finite element models indicates that optimized parameters significantly enhance system efficiency. In Contribution 4, an energy harvesting system is proposed to collect downward airflow from helicopters or multi-axis unmanned rotary-wing aircraft. This wind force drives a magnet to rotate, generating a repulsive force that causes a double elastic steel system to slap and vibrate periodically, generating more electricity than traditional systems. In Contribution 5, a parameter identification method for the multiparameter identification study of a linear-arch composite beam piezoelectric energy harvester is proposed. An experimental platform verifies that the method has high accuracy and practicability. In Contribution 6, optimized cantilever geometries developed using the design of experiments approach are analyzed and combined with frequency up-conversion excitation to convert random kinetic ambient motion into periodic excitation of the harvester. In Contribution 7, piezoelectric energy harvesting is studied in the context of a low-velocity impact of a rigid mass on a composite beam. The methodology includes modeling the open-circuit impact response in a finite element package, formulating a lumped parameter model for the piezoelectric transducer connected to the harvesting circuit, and experimentally verifying the impact using a custom portable configuration with impactor motion control. In Contribution 8, a quantitative comparison of three passive rectifiers in a low-power, low-voltage electromagnetic energy harvesting subsystem is presented. In Contribution 9, a novel method to increase the bandwidth of a cantilever beam by using an embedded transverse out-of-plane movable mass is presented. The concept is investigated through experimentation with a movable mass, in the form of a solid sphere, embedded within a stationary proof mass with hollow cylindrical chambers. 

In Contribution 10, flexible generators based on lead-free barium titanate (BaTiO_3_) and a polydimethylsiloxane (PDMS) polymer were developed. Through a comparative study, the authors investigate the impact of multi-walled carbon nanotubes (MWCNTs) through structural, morphological, electrical, and electromechanical measurements. In Contribution 11, the authors report for the first time a composite of ferroelectric antimony sulfoiodide (SbSI) nanowires and non-ferroelectric titanium dioxide (TiO2) nanoparticles as a pyroelectric nanogenerator. In Contribution 12, the authors propose a novel flexible triboelectric nanogenerator (FTENG) using a flexible micro-needle-structured polydimethylsiloxane (MN-PDMS) in combination with comfortable, commercially available 2D-polyester fibers and electroless nickel-plated cotton cloth, both of which are widely used in daily life. 

In Contribution 13, the authors present a simulation of an environment monitoring device powered by a thermoelectric generator (TEG) that harvests energy from the temperature difference between air and soil. In Contribution 14, an energy harvesting system for solar energy is presented, featuring a flexible battery, a semi-flexible solar harvester module, and a Bluetooth Low Energy (BLE) microprocessor module.

In Contribution 15, the authors model and optimize various components of a Radiofrequency (RF) Energy Harvesting (EH)-assisted, QB-enabled Internet of Things (IoT) system. In Contribution 16, a new RF Energy Harvesting (RF-EH) system designed for Wireless Sensor Network (WSN) feeding is proposed. This system utilizes two different monitored architectures controlled by switch circuits based on input power levels. In Contribution 17, the authors propose a self-threshold voltage compensated Radio Frequency to Direct Current (RF-DC) converter that operates at 900 MHz and 2.4 GHz for RF energy harvesting applications. In Contribution 18, the authors present a multiband ambient RF energy harvester equipped with a high-gain wideband circularly polarized antenna, aimed at enabling self-powered wireless sensors. In Contribution 19, the authors introduce a metamaterial (MTM)-integrated high-gain rectenna for RF sensing and energy harvesting applications, operating at the 2.45 GHz ISM band. The novel MTM superstrate approach with a three-layered integration method is presented for the first time for rectenna applications.

In Contribution 20, the authors introduce a multiple concurrent slotframe scheduling (MCSS) protocol for wireless power transfer (WPT)-enabled wireless sensor networks. Simulation results from their study indicate that MCSS outperforms the traditional TSCH medium access control protocol and TSCH multiple slot frame scheduling (TMSS) in terms of average end-to-end delay, aggregate throughput, and average harvested energy. In Contribution 21, the authors discuss an Internet of Things (IoT)-enabled smart meter that utilizes energy-efficient simultaneous wireless information and power transfer (SWIPT) for a wireless-powered smart grid communication network. In Contribution 22, the detection problem is tackled using an optimal stopping framework, dealing with highly unstable likelihoods due to noisy measurements. In Contribution 23, the Bluetooth Low Energy (BLE) specification introduces support for Low Power Nodes (LPNs) via the friendship feature, where an LPN pairs with a neighboring friend node. In Contribution 24, the authors explore the LIoT concept by designing, implementing, and evaluating an LIoT node’s communication and energy harvesting performance. In Contribution 25, an innovative wireless monitoring system for brake system diagnostics is presented. Due to the difficulty of applying a wired monitoring system to a freight convoy, a low-power system architecture focusing on energy harvesting and wireless communication was developed. In Contribution 26, a residual energy estimation-based medium access control (REE-MAC) protocol for wireless powered sensor networks (WPSNs) is presented. In Contribution 27, the energy consumption of popular IoT wireless technologies such as Sigfox, LoRaWAN, NB-IoT, Wi-Fi, and BLE is analyzed. Smart meters’ energy consumption is broken down into metering, standby, and communication processes. In Contribution 28, the authors discuss how wireless-powered communication networks (WPCNs) will enable massive machine-type communications (MTCs), a significant service domain for 5G and beyond systems. In Contribution 29, the focus is on distributed transmit power control for energy-efficient wireless-powered secure communications, improving sum secrecy energy efficiency through distributed algorithms. In Contribution 30, the authors examine energy-efficient wireless-powered secure communications involving multiple sets of transmitters, receivers, and energy harvesting nodes. 

In Contribution 31, possible aging mechanisms are reviewed, elaborating on the effect of aging on the electrical performance of e-textiles. The review provides an overview of various laboratory methods for investigating accelerated functional aging. Finally, a model of cumulative fatigue damage theory is proposed to model the changes in e-textile properties over their lifetime.

The above studies collectively contribute to advancing the fields of energy harvesting and IoT technologies, showcasing innovative approaches, practical applications, and theoretical insights that pave the way for sustainable and autonomous sensor networks. We extend our gratitude to all of the authors and reviewers whose contributions have enriched this Special Issue.
